# 体外循环在局部晚期肺恶性肿瘤切除手术中的应用体会

**DOI:** 10.3779/j.issn.1009-3419.2011.03.14

**Published:** 2011-03-20

**Authors:** 永 栾, 庆伟 谭, 晓明 卞

**Affiliations:** 1 116011 大连，大连医科大学附属第一医院麻醉科 Department of Anesthesiology, the First Affiliated Hospital of Dalian Medical University, Dalian 116011, China; 2 116011 大连，大连医科大学附属第一医院胸外科 Department of Thoracic Surgery, the First Affiliated Hospital of Dalian Medical University, Dalian 116011, China; 3 116011 大连，大连医科大学附属第一医院心外科 Department of Cardiac Surgery, the First Affiliated Hospital of Dalian Medical University, Dalian 116011, China

**Keywords:** 肺肿瘤, 体外循环, 手术, Lung neoplasms, Cardiopulmonary bypass, Surgery

## Abstract

**背景与目的:**

体外循环（cardiopulmonary bypass, CPB）应用于侵犯心脏及大血管的肺局部晚期恶性肿瘤切除手术开展较少。本研究回顾分析我院近年来完成的CPB辅助下肺局部晚期恶性肿瘤切除手术，总结CPB的应用管理体会。

**方法:**

CPB辅助下完成肺局部晚期恶性肿瘤切除手术4例，临床分期均为T4N0M0，非CPB下均无法根治性切除。4例均采用浅低温体外循环方法，转流时间为79 min-155 min，心脏停跳时间57 min-126 min。

**结果:**

本组无术中死亡。CPB转流平稳，均自动复跳。4例停机顺利。全组均顺利出院，1例术后13个月因心衰死亡，1例术后22个月死于肿瘤远处转移。1例术后19个月、1例术后5个月仍存活。

**结论:**

CPB有助于帮助外科医生对局部晚期肺肿瘤施行完全性肺癌切除。

## Abstract (en)

**Background and objective:**

Cardiopulmonary bypass (CPB) in the treatment of locally advanced pulmonary malignant tumors invaded heart or great vessels was not well underwent. The aim of this study is to report the results of these cases with locally advanced pulmonary malignant tumor which were completely resected under CPB in our hospital.

**Methods:**

Four patients with locally advanced pulmonary malignant tumors were operated under CPB. All patients had stage Ⅲb disease (T4N0M0). Moderate hypothermia CPB was used for all 4 cases. The CPB time was 79 min-155 min and aortic cross-clamp time was 57 min-126 min, respectively.

**Results:**

There was no operative death in this series. CPB was smooth, and heart automatically rebeated in the 4 patients. All the 4 patients were discharged in 3 weeks. One patient dead of heart failure at 13 months after operation, one case dead of distant tumor metastasis at 22 months after operation. The other two patients have survived over 25 months and 11 months, respectively.

**Conclusion:**

CPB is helpful for completely removal of locally advanced pulmonary malignant tumors.

肺癌侵犯心脏、大血管无论在屍解，还是外科临床手术中均非少见。过去对这类病变均视为外科禁忌症，而且大多数病变用常规的手术方法不可能切除病变。对于这类患者是否适合外科手术治疗，过去一直存在争议。然而，经近30多年来国内外的研究^[1-6]^发现，对于这些局部晚期的肺癌患者，有相当多的患者并无远处转移，如果能将肺癌连同受侵的心脏大血管施行完全性切除术，许多患者能获得很好的预后和长期生存率。肺癌侵犯心脏大血管者，一部分病变通过借助于一些血管器械，就能达到肺癌和受侵组织器官完全切除，而有一部分则需要在体外循环（cardiopulmonary bypass, CPB）下才能施行肺癌的扩大切除术^[1-12]^。

最早将CPB技术应用于肺癌手术始于20世纪60年代。1967年，意大利医生Ruggieri首先在CPB下施行肺切除合并部分左心房切除术治疗肺癌侵犯左心房的T4肺癌，以后日本、美国等国的胸外科医生相继开展了此项手术^[2]^。国内周清华^[4-6, 8-12]^从20世纪80年代初开始施行此类手术，先后应用CPB技术施行肺切除合并上腔静脉切除重建、部分左心房切除重建、部分胸主动脉切除重建术，左心房、右心房肺癌癌栓摘除术等肺癌手术中，获得较好的近期和远期效果。目前，国内外均有部分大的医疗中心将CPB技术用于肺癌手术，并逐渐被更多的医院采用^[1, 7, 13-16]^。

CPB应用于侵犯心脏及大血管的肺局部晚期恶性肿瘤切除手术开展较少。一方面是因为人们担心CPB引起的相关并发症，如全身肝素化导致术中或术后的大出血、插管和转流导致瘤体脱落引起肿瘤转移或播散等。另一方面，人们认为既然肿瘤侵及大血管，已属晚期，预后不佳，手术切除意义不大^[1]^。尽管如此，根据国内、外报道，对于肺局部晚期恶性肿瘤，尤其是已侵及心脏、大血管，常规方法切除风险巨大的病例，CPB为该类肿瘤的完全性切除提供了新的平台，并能明显改善病人预后。Horita等^[15]^报道1例侵及胸主动脉的肺癌于CPB下行肺癌根治术，随访5年生存良好；Vaporciyan等^[16]^报告19例胸腔内肿瘤切除，其中17例肿瘤侵犯心脏大血管，需要在体外循环下施行根治性切除，最长随访27个月，1、2年的生存率分别为65%和45%。周清华等^[8]^报道肺癌侵犯左心房的扩大切除者中生存最长的病例已达10年以上。我院近年来完成CPB辅助下肺局部晚期恶性肿瘤切除手术4例，现将报告如下。

## 1 临床资料

### 1.1 一般资料

2007年3月-2010年5月，我院在CPB辅助下完成肺局部晚期恶性肿瘤切除手术4例。其中男性3例，女性l例；年龄38岁-64岁；体重47 kg-65 kg。其中右肺中心型肺癌2例，临床分期均为T4N0M0；原发性右肺门恶性神经鞘瘤1例，此3例心功能均Ⅰ级；原发性右下肺静脉成骨性骨肉瘤1例，心功能Ⅲ级。4例均为右肺门巨大肿块侵及右肺动脉主干、肺静脉和左心房，其中2例肿瘤突入左心房，2例侵及上腔静脉致阻塞合并上腔静脉综合征，非CPB下均无法根治性切除。

### 1.2 手术方法

手术采用胸骨正中切口1例，右胸后外侧切口3例。4例均行右全肺切除淋巴结清除同时加部分左心房切除重建，2例同时施行上腔静脉人工血管置换术。

### 1.3 体外循环方法

4例均采用浅低温（鼻咽温度31 ℃-32 ℃）体外循环方法，流量2.0 L/min-2.4 L/min，体外循环中维持平均动脉压60 mmHg-80 mmHg。均采用4:1含血冷晶体停跳液心肌保护。采用JOSTRA-Ⅱ型人工心肺机，TERUMO-18型膜式氧合器。预充液为乳酸钠林格氏液和琥珀酰明胶各1, 000 mL，转中加入甲强龙15 mg/Kg、乌斯他丁10, 000 u/kg。体外循环中常规超滤。转流时间为79 min-155 min，心脏停跳时间57 min-126 min。动、静脉插管为升主动脉、上腔静脉和下腔静脉2例，升主动脉，右心房1例，股动脉和右心房插管1例。

## 2 结果

本组无术中死亡。CPB均转流平稳，心脏均自动复跳。4例停机顺利，其中1例停机后气管插管内见淡粉色泡沫样痰，残肺可闻及水泡音，SPO_2_降至90%。经充分吸痰，并给予强心、利尿后，泡沫样痰及水泡音均消失，SPO_2_升至97%-99%。全组均顺利出院，1例术后13个月因心衰死亡，1例术后22个月死于肿瘤远处转移。1例术后19个月、1例术后5个月仍存活。

## 3 讨论

国内外文献^[8-16]^报道应用体外循环技术治疗侵犯心脏大血管的局部晚期非小细胞肺癌，能明显改善患者预后，并使20%-30%的患者获得长期生存。由于本组病例相对较少，对体外循环辅助下的肺局部晚期恶性肿瘤切除手术是否能够明显改善预后、CPB插管和转流能否导致肿瘤转移扩散等疑问，尚难得出明确结论。我们仅对CPB的应用体会加以讨论。首先，可在CPB建立前充分探察，清楚地了解心脏、大血管的受侵范围。能够在非CPB下完成的操作尽量在转流前进行，涉及大血管的操作可在CPB插管完成后施行，必要时可随时转流。这样，CPB建立后可以很快切除病灶，即有利于充分暴露术野，使心脏、大血管的修补成形易于进行，又使体外循环的转机时间尽可能缩短^[15]^。本组病例术前经充分病情评估，并预先有针对性地设计了CPB的建立方法，故CPB建立均较顺利，并最大限度的减少了转流时间。

其次，应合理有效地建立CPB。与心脏外科建立CPB插管不同，肺局部晚期恶性肿瘤手术CPB插管变异较大，需根据病变部位的不同而采取不同的方式。全肺切除手术一般采取胸部后外切口，心脏、血管插管操作部位较深，加之肿瘤巨大或伴有实变的肺而使术野显露不佳，本组4例中就有3例采用了后外切口。解剖关系，左侧开胸时动静脉插管较右侧开胸困难，本组病例均恰巧为右胸肿瘤，插管相对容易^[2]^。Korst等^[14]^认为，对于肿瘤较大的患者最好行股动、静脉插管。肿瘤未侵及右房或腔静脉时，可采取经右房插管，如已累及则应行上下腔静脉插管。当无法摆左心引流时，为防止心脏过涨，可考虑经肺动脉插管引流。当肿瘤未累及左心系统时，可考虑并行循环下手术。本组病例均累及左房，为防止气栓，4例手术均在主动脉阻断下完成。

另外，本组一例停机后出现肺水肿，考虑与一侧全肺切除后，肺循环将由双侧改为单侧，同时CPB血液稀释有关。因此，主动脉开放后，应控制并逐渐恢复流量，同时适当提高胶体渗透压（利尿或超滤），停机过程缓慢还血，逐步停机，防止出现残余单肺肺水肿。最后，为尽量减少CPB引起的肺损伤，可在转中给予适量抗炎药物，如乌司他丁、糖皮质激素等，以减少炎症反应^[7]^。
